# Evaluating the efficacy of *Ocimum tenuiflorum* as a therapeutic agent in colorectal cancer

**DOI:** 10.3389/fonc.2026.1867252

**Published:** 2026-07-08

**Authors:** Aishwarya H. K., Shamanth D. Harishkumar, Pruthvi H. S. Gowda, Chandan Shivamallu, Shiva Prasad Kollur, Chandan Dharmashekar, Sumitha Elayaperumal

**Affiliations:** 1 Department of Microbiology, JSS Academy of Higher Education & Research, Mysuru, Karnataka, India; 2 Department of Biotechnology and Bioinformatics, JSS Academy of Higher Education & Research, Mysuru, Karnataka, India; 3 School of Physical Sciences, Amrita Vishwa Vidyapeetham, Mysuru, Karnataka, India

**Keywords:** colorectal cancer, cytotoxicity, molecular docking, molecular dynamics simulation, natural anticancer compounds, *Ocimum tenuiflorum*

## Abstract

**Background:**

Colorectal cancer (CRC) remains a leading cause of cancer-related morbidity and mortality worldwide, with persistent challenges of systemic toxicity, chemoresistance, and limited efficacy of conventional therapies underscoring the need for safer therapeutic alternatives. *Ocimum tenuiflorum* (Holy Basil), a phytochemically rich medicinal herb with established antioxidant, anti-inflammato

**Methods:**

An integrated *in vitro* and *in silico* approach was employed. The ethanolic extract of *O. tenuiflorum* leaves was prepared by Soxhlet extraction and evaluated for cytotoxicity (MTT, XTT, LDH release, Trypan Blue exclusion) against HCT116 colorectal cancer cells, antioxidant activity (DPPH, ABTS, FRAP assays), and anti-inflammatory activity (nitric oxide and cyclooxygenase inhibition assays) using RAW 264.7 macrophages. Computational analyses included protein-protein interaction (PPI) network construction via STRING, molecular docking (Glide SP/XP) of bioactive ligands against NF-κB (PDB: 1NFK) and JAK1 (PDB: 5XGJ), a 200 ns molecular dynamics (MD) simulation of the lead complex, and Density Functional Theory (DFT) calculations at the B3LYP/6-31G* level.

**Results:**

The extract exhibited significant, concentration-dependent cytotoxicity against HCT116 cells (MTT IC_50_: 70.4 µg/mL), substantial antioxidant activity (DPPH IC_50_: 34.7 µg/mL), and notable inhibition of nitric oxide and COX activity, indicating modulation of oxidative stress and tumor-associated inflammation. PPI analysis revealed high-connectivity NFKB1 and PIK3CA signaling hubs central to CRC progression. Among all screened phytochemicals, rosmarinic acid demonstrated the highest binding affinity against NF-κB (docking score: −5.898 kcal/mol) and JAK1 (−6.812 kcal/mol), forming a stable complex over the 200 ns MD simulation (backbone RMSD: ±2.7 Å). DFT analysis revealed a moderate HOMO–LUMO gap (3.895 eV), indicating favorable electronic stability and chemical reactivity.

**Discussion:**

The crude ethanolic extract of *O. tenuiflorum* demonstrated consistent, multi-target bioactivity, combining antioxidant, anti-inflammatory, and cytotoxic effects, supporting its potential as a chemopreventive and therapeutic agent against CRC. Within this extract, rosmarinic acid emerged as the principal bioactive contributor, exhibiting markedly higher binding affinity than all other screened phytochemicals and the reference standard doxorubicin at both NF-κB and JAK1 targets, with stable complex formation confirmed over 200 ns of MD simulation. These findings position *O. tenuiflorum*, and rosmarinic acid specifically, as a promising, safer alternative to conventional chemotherapy. Future studies should focus on in vivo validation, bioavailability-enhancing nanoformulation strategies, and clinical evaluation to translate these findings toward adjunctive cancer therapy.

## 1 Introduction

Colorectal cancer (CRC) is one of the most common malignancies worldwide, accounting for approximately 10% of global cancer incidence and mortality (1). According to GLOBOCAN 2022, there were approximately 1.93 million new CRC cases and over 904,000 deaths globally, with incidence projections indicating a continued rise through 2050, driven by aging populations and westernized lifestyle factors. It ranks as the third most commonly diagnosed cancer in men and the second in women, with significant geographic variation in incidence rates, reflecting differences in diet, lifestyle, and healthcare access. CRC develops from the epithelial cells lining the colon or rectum, primarily through the adenoma-carcinoma sequence, which involves the progressive accumulation of genetic and epigenetic factors. These changes will lead to normal mucosa becoming adenomatous polyps and, ultimately, invasive carcinoma (2). The risk factors for CRC are complex, as they include both non-modifiable and modifiable risk factors. Non-modifiable risk factors for CRC increase with age, the vast majority of cases occur in persons over 50 years old, and a family history of CRC and hereditary syndromes, such as Lynch syndrome and familial adenomatous polyposis (FAP), are considered to be significant non-modifiable risk factors. Modifiable risk factors are largely related to lifestyle and dietary practices. There is evidence that high consumption of red and processed meats, low fiber intake, obesity, physical inactivity, smoking, and heavy alcohol use increase the risk of CRC (3). On the other hand, diets high in fruits, vegetables, and whole grains and engaging in regular physical activity appear to reduce risk. CRC is a result of the accumulation of genetic and epigenetic changes, leading to the disturbance of pivotal cellular and signal transduction pathways that normally govern at least three principles of cellular functions like growth, differentiation and apoptosis. Chronic inflammation is widely recognized as a critical factor in the initiation and progression of Colorectal Cancer (4). Among the key molecular mechanisms involved, the NF-κB signaling pathway plays a central role in regulating genes associated with inflammation, cell survival, proliferation, and tumor progression. Persistent activation of NF-κB signaling in colorectal epithelial cells leads to increased expression of pro-inflammatory cytokines, anti-apoptotic proteins, and growth factors, thereby promoting tumor development and resistance to apoptosis. In parallel, the JAK-STAT signaling pathway is frequently activated by cytokines and growth factors within the tumor microenvironment, resulting in phosphorylation and activation of STAT transcription factors that regulate genes involved in cellular proliferation, immune modulation, and survival (5). Dysregulation of JAK-STAT signaling has been implicated in colorectal tumorigenesis, where sustained activation contributes to enhanced tumor cell proliferation, immune evasion, and chronic inflammatory signaling. Collectively, aberrant activation of NF-κB and JAK-STAT pathways creates a pro-tumorigenic environment that supports colorectal cancer initiation, progression, and resistance to programmed cell death (6). Overall, it is apparent that despite advances in CRC prevention, screening, and treatment modalities, the development of new and effective therapy has been limited due to multiple factors, including the fact that relatively effective surgical resection is commonly the first treatment modality for patients with early-stage CRC, but diminishingly effective in the advanced or metastatic settings. In advanced CRC chemotherapy regimens such as FOLFOX and FOLFIRI are widely used to treat advanced CRC but have severe side effects including neurotoxicity, myelosuppression, gastrointestinal side effects, and fatigue (7). They also develop chemoresistance, limiting their long-term use. Other targeted therapies, such as bevacizumab (anti-VEGF) and cetuximab (anti-EGFR), have provided patients with improved outcomes, however they are limited based on individual genetic profiles, high costs, and toxicity effects such as hypertension and dermatological side effects (8). Immunotherapy checkpoint inhibitors have been very potent in microsatellite instability-high (MSI-H) tumors; however, they will not be efficacious in the majority of CRC patients which are associated with microsatellite-stable (MSS) tumors (9). Given that CRC therapies face these challenges, there remains a substantial unmet clinical need to identify novel, safer, and more efficacious therapeutic strategies. Phytotherapy is the use of plant-derived natural compounds as therapeutic agents represents a compelling and historically validated approach to cancer management.

**Figure 1:**
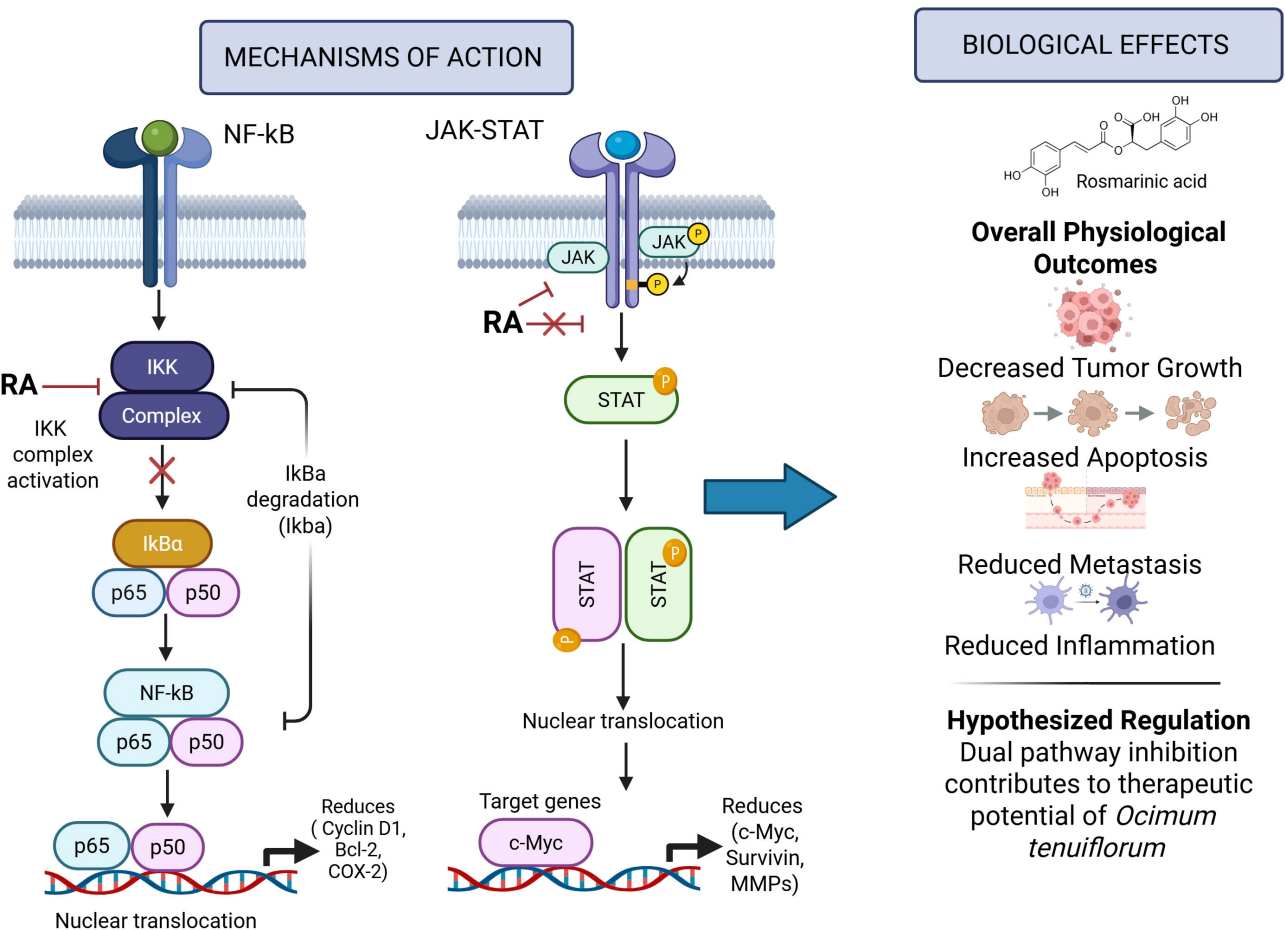
Proposed mechanism of rosmarinic acid–mediated anticancer activity through NF-κB and JAK–STAT pathway modulation in colorectal cancer.

A natural product of exceptional therapeutic promise is *Ocimum tenuiflorum* L. (Holy Basil or Tulsi, family Lamiaceae), a medicinal herb with a long history of use in Ayurvedic and traditional medicine systems across South and Southeast Asia. *O. tenuiflorum* is phytochemically rich, containing a diverse array of bioactive constituents including eugenol, ursolic acid, rosmarinic acid, apigenin, luteolin, and β-caryophyllene, which collectively confer broad-spectrum pharmacological activity encompassing antioxidant, anti-inflammatory, immunomodulatory, and anticancer properties (10). Several preclinical studies have demonstrated the anticancer activity of *O. tenuiflorum* across multiple cancer models, providing an important scientific foundation for the present investigation. Lam et al. (2017) reported that *O. tenuiflorum* leaf extracts exhibited significant cytotoxic activity against MCF-7 breast cancer cells, with inhibition attributed to the induction of apoptosis and cell cycle arrest, highlighting the pro-apoptotic potential of its bioactive constituents (11). Similarly, Moyo et al. (2024) demonstrated cytotoxic activity of crude *O. tenuiflorum* extracts against the A375 malignant melanoma cell line, with the ethanolic extract displaying the most potent antiproliferative effects, supporting the solvent-dependent bioactivity of the plant (12). Shaikh (2025) conducted a comparative evaluation of *O. tenuiflorum* and *Catharanthus roseus*, reporting significant antioxidant and anticancer activity for *O. tenuiflorum* extracts, further validating its potential as a source of bioactive anticancer compounds (13). A comprehensive phytochemical characterization of *O. tenuiflorum* by Bhattarai et al. (2024) confirmed the pharmacological relevance of its major bioactive secondary metabolites including rosmarinic acid, oleanolic acid, luteolin, ursolic acid, and limonene demonstrating antioxidant, neuroprotective, anticancer, and anti-inflammatory properties (14). The preclinical anticancer mechanisms described include induction of apoptosis, inhibition of angiogenesis, modulation of inflammatory cytokine levels, and suppression of NF-κB activation, providing molecular-level support for the therapeutic rationale of the present investigation. Recent clinical and preclinical evidence by Liu et al. (2025) directly demonstrated that rosmarinic acid suppresses NF-κB signaling in CRC cell lines, leading to reduced proliferation and increased apoptosis, establishing an independent mechanistic basis for the computational targets selected in the current study (15). Therapeutic applications of rosmarinic acid in overcoming chemoresistance and attenuating chemotherapy-induced toxicity have been documented by Ballesteros-Tapias et al. (2024), underscoring its multifaceted pharmacological profile and its promise as an adjunctive oncological agent (16). Despite this growing body of evidence, critical gaps remain in the literature. No prior study has systematically investigated the anticancer activity of *O. tenuiflorum* specifically against colorectal cancer cell lines in conjunction with a comprehensive computational analysis of its bioactive constituents against validated CRC molecular targets. The specific interactions of *O. tenuiflorum* phytochemicals with proteins governing the NF-κB and JAK-STAT signaling pathways both centrally implicated in colorectal tumorigenesis has not been characterized through molecular docking and molecular dynamics simulations. The electronic and drug-likeness properties of its key constituents have similarly remained unexamined through Density Functional Theory (DFT) analysis. The present study was therefore designed to address these gaps through an integrated *in vitro* and *in silico* approach. *In vitro* cytotoxicity, antioxidant, and anti-inflammatory assays were performed using the HCT116 colorectal cancer cell line to quantify the biological activity of the *O. tenuiflorum* ethanolic extract. Concurrently, *in silico* analyses encompassing protein-protein interaction (PPI) network construction, molecular docking, molecular dynamics simulations, and DFT calculations were conducted to identify the principal bioactive contributor and characterize its molecular interactions with key CRC-associated target proteins (PDB IDs: 1NFK and 5XGJ). By integrating experimental and computational evidence, this study provides the first systematic evaluation of *O. tenuiflorum* as a phytotherapeutic candidate for colorectal cancer, establishing a scientific rationale for its further preclinical and clinical development. (**Figure 1**).

## 2 Materials and methods

### 2.1 Extraction and preparation of *Ocimum tenuiflorum* extract

Fresh leaves of *Ocimum tenuiflorum* L. (Holy Basil, family Lamiaceae) were collected from the JSS Academy of Higher Education and Research, University campus, Mysuru, Karnataka, India, and taxonomically authenticated by Prof. V. Biligirimanga, PG Department of Botany, JSS College of Arts, Commerce & Science, Mysore (Authentication Ref. No.: JSSCM/PGBOT/2026-27/001). The collected leaves were thoroughly washed with distilled water to remove surface contaminants and air-dried at room temperature in a shaded, well-ventilated area, away from direct sunlight, to preserve the phytochemical integrity of the plant material. The dried leaves were subsequently ground into a fine homogeneous powder using a mechanical grinder. Soxhlet extraction was performed using 95% ethanol as the solvent for approximately 8 hours to ensure exhaustive extraction of bioactive compounds. The resultant extract was filtered through Whatman No. 1 filter paper to eliminate particulate residues. The filtered extract was concentrated under reduced pressure using a rotary evaporator maintained at 40 °C to remove the solvent completely, yielding a thick viscous extract. The concentrate was further dried under reduced pressure to obtain a solid crude extract. A total yield of 346.51 mg of crude extract was obtained from 25 g of powdered plant material, corresponding to an extraction yield of approximately 1.39% (w/w). The solid extract was stored at −20 °C in sealed vials until further use. Prior to experimental use, the extract was reconstituted in dimethyl sulfoxide (DMSO) to prepare concentrated stock solutions, which were subsequently diluted in appropriate culture media to achieve the desired working concentrations for *in vitro* cell treatment assays. The final DMSO concentration in all treatment conditions was maintained at ≤0.1% (v/v) to eliminate any potential solvent-induced cytotoxic effects. To ensure experimental consistency and eliminate batch-to-batch variability as a confounding factor, all *in vitro* assays were performed using extract derived from a single homogeneous batch prepared under the standardized conditions described above (17).

### 2.2 Cell lines

The colorectal cancer cell line HCT116 (CCL-247) was obtained from the American Type Culture Collection (ATCC) and used as the primary experimental model in this study. HCT116 was selected on the basis of its well-characterized molecular profile including microsatellite instability (MSI-H), constitutive NF-κB and JAK-STAT pathway activation, and activating KRAS mutation, which renders it directly relevant to the signaling targets investigated computationally in this study. Cells were cultured in Dulbecco’s Modified Eagle Medium (DMEM) supplemented with 10% fetal bovine serum (FBS) and 1% penicillin-streptomycin, and maintained in a humidified atmosphere with 5% CO_2_ at 37 °C. Cells were continuously sub-cultured and monitored for contamination, and all experiments were performed using cells within 20 passages to ensure consistency and reliability (18).

### 2.3 Antioxidant assays

#### 2.3.1 DPPH radical scavenging assay

The antioxidant activity of *Ocimum tenuiflorum* extract was determined using a number of assays. To determine the DPPH radical scavenging activity, 100 µL of the different concentrations of the extract were added to 100 µL of 0.1 mM DPPH solution in methanol in a 96-well plate, and the mixture was incubated in the dark for 30 minutes at room temperature (19). After the incubation period, the decrease in absorbance was measured at 517 nm with a microplate reader and the DPPH radical scavenging activity was determined (20).

#### 2.3.2 ABTS radical cation decolorization assay

The ABTS radical cation decolorization assay involved generating the ABTS radical cation (ABTS^+^) by mixing 7 mM ABTS solution with 2.45 mM potassium persulfate and incubating it in the dark at room temperature for 12–16 hours. The ABTS^+^ solution was then diluted with ethanol to an absorbance of 0.70 ± 0.02 at 734 nm. 100 µL of the extract at different concentrations were added to 100 µL of the ABTS^+^ solution in a 96-well plate. After 6 minutes of incubation at room temperature, the absorbance was measured at 734 nm, and the antioxidant activity was determined by the reduction in absorbance (20).

#### 2.3.3 Ferric reducing antioxidant power assay

The ferric reducing antioxidant power (FRAP) assay was performed in a 96-well plate by adding 300 µL of FRAP reagent which is made up of 300 mM acetate buffer (pH 3.6), 10 mM TPTZ solution, and 20 mM ferric chloride solution to 10 µL of the extract. The mixture was left to incubate at 37 °C for 30 minutes. Once the incubation was complete the increase in the absorbance was taken at 593 nm. The antioxidant capacity of the extract was determined by comparison to a standard curve of ferrous sulfate (21).

### 2.4 Anti-inflammatory assays

#### 2.4.1 Nitric oxide assay

The anti-inflammatory activity of *Ocimum tenuiflorum* extract was evaluated using multiple *in vitro* assays. Nitric oxide (NO) production was assessed using RAW 264.7 murine macrophages, which were seeded in 96-well plates at a density of 1 × 10^5^ cells per well and incubated overnight to allow cell adherence. The cells were pretreated with specified concentrations of the extract for 1 h, followed by stimulation with lipopolysaccharide (LPS, 1 µg/mL) for 24 h. To quantify NO production, 50 µL of culture supernatant was mixed with 50 µL of Griess reagent (1% sulfanilamide and 0.1% N-(1-naphthyl) ethylenediamine dihydrochloride in 2.5% phosphoric acid) in a 96-well plate. After incubation at room temperature for 10 min, absorbance was measured at 540 nm using a microplate reader. Nitrite concentrations, representing stable NO metabolites, were calculated using a sodium nitrite standard curve (22).

#### 2.4.2 Cyclooxygenase inhibition assay

The Cyclooxygenase (COX) Inhibition Assay was conducted using a COX inhibition screening kit. The assay measured the inhibition of COX-1 and COX-2 enzyme activities by the extract. The reaction mixture consisted of buffer solution, heme, enzyme (COX-1 or COX-2), and the extract at different concentrations. The reaction was started by adding arachidonic acid substrate as per kit instruction, and incubated at 37 °C for the specified time. The production of prostaglandin G2 (PGG2) was measured by absorbance at 450 nm using a microplate reader. The percentage inhibition of COX activity was calculated in relation to the control reactions without extract (23).

### 2.5 Cytotoxicity assays

#### 2.5.1 MTT assay

The extract of *Ocimum tenuiflorum* was assessed for cytotoxic effects according to several established assays. For the MTT assay, cancer cells were plated in 96-well plates at a density of 5,000 cells per well and kept for overnight adherence. After treatment of the extract at various concentrations for 24 hours, 10 µL of MTT (5 mg/mL in PBS) were added to each well and incubated for 4 hours at 37 °C. The formazan crystals were dissolved in 100 µL of dimethyl sulfoxide (DMSO), a microplate reader was used to measure the absorbance of each well at 570 nm to assess cell viability (12).

#### 2.5.2 XTT assay

The XTT assay was done similarly, where the cells were seeded in 96-well plates and extracted. After 24 hours of treatment, each well was supplemented with 50 µL of XTT reagent mixed with the electron coupling reagent and incubated for 4 hours. The color change as a result of cell viability was documented at 450 nm using a plate reader (24).

#### 2.5.3 LDH release assay

The LDH release assay was performed in 96-well plates and treated with the extract. After 24 hours, the supernatant from each well was collected and transferred to a new 96-well plate. LDH activity was measured using a commercially available LDH assay kit (e.g., Roche) according to the manufacturer’s instructions. The absorbance was read at 490 nm, indicating the amount of LDH released due to membrane damage, thus reflecting cytotoxicity (25).

### 2.6 Cell viability assays

#### 2.6.1 Trypan blue exclusion assay

The Trypan Blue exclusion assay involved seeding cells in 6-well plates and treating them with the extract for 24 hours. Following treatment, cells were harvested and stained with 0.4% Trypan Blue solution. Viable (unstained) and non-viable (blue-stained) cells were counted using a hemocytometer under a light microscope. The percentage of cell viability was calculated to assess the cytotoxic effects of the extract (19).

### 2.7 Statistical methods

Statistical analyses were performed using GraphPad Prism software (version 8.0). All experiments were conducted in triplicate, and the results are presented as mean ± standard deviation (SD). Data normality was assessed using the Shapiro–Wilk test, which confirmed that the data were normally distributed. Statistical significance among groups was determined using one-way analysis of variance (ANOVA) followed by Tukey’s *post hoc* test for multiple comparisons. A p-value of less than 0.05 was considered statistically significant (21).

### 2.8 Computational analysis

#### 2.8.1 Protein-protein interaction network analysis

SRING 12.0 software is used for protein-protein interaction (PPI) network analysis, which identifies and examines the interactions between the proteins in a biological system. To construct the network using STRING, unconnected nodes were eliminated, and interaction scores less than 0.4 were selected.

As sources of active interaction, databases, gene fusion, neighborhood, experiments, co-occurrence, co-expression, and text mining were used to form the network (26).

#### 2.8.2 Ligand preparation

Three-dimensional structures of the bioactive ligands from *Ocimum tenuiflorum* (OT) leaf were obtained from IMPPAT database and processed using the LigPrep module in the Schrödinger suite, incorporating energy minimization to generate optimized low-energy conformations. All the compounds were subjected to molecular docking against the selected target proteins to enable objective, comparative evaluation of binding affinities. Among all the docked compounds Rosmarinic acid was subsequently identified as the lead compound based on its superior Glide docking scores against both target proteins (1NFK and 5XGJ) relative to all other screened constituents, consistent with its established mechanistic relevance to NF-κB and JAK-STAT pathway suppression in colorectal cancer. This data-driven selection confirmed by the docking results presented in **Table 1** further validates the prioritization of rosmarinic acid for downstream molecular dynamics simulation and DFT analysis (27).

**Table 1:** 2D interaction diagram of the Ligands with their interactions with the targeted proteins PDB (ID: 1NFK, 5XGJ), (a) Standard- Doxorubicin, (b) Ligand-IMPHY004597.

Protein-Ligand	Two-dimensional inter-molecular interaction	Docking score	Active site residues
1NFK-31703	A 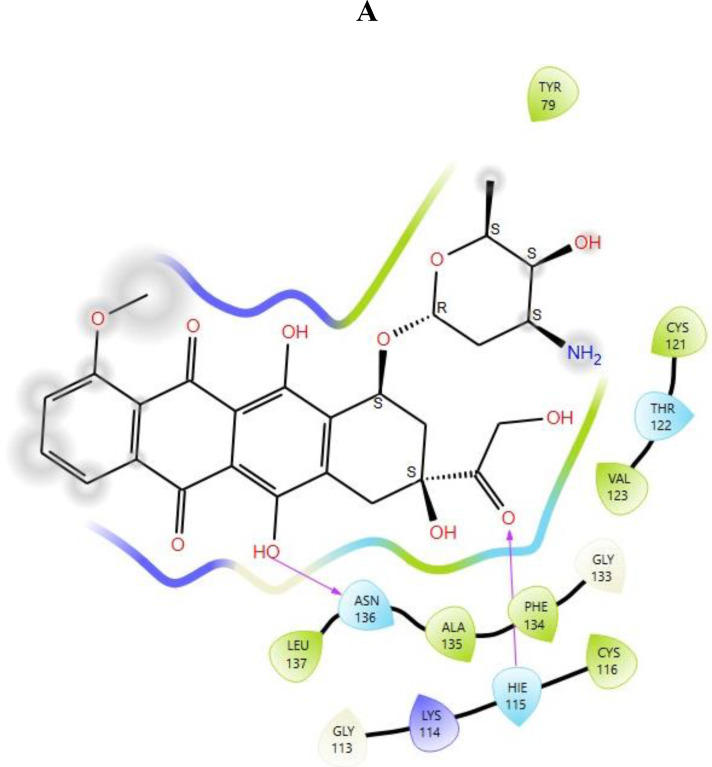	-3.286	HIE 115, ASN 136
1NFK - IMPHY004597	B 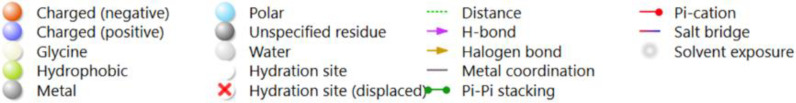	-5.898	ASN 136, ALA 135, LYS 114, HIE 115
5XGJ-31703	A 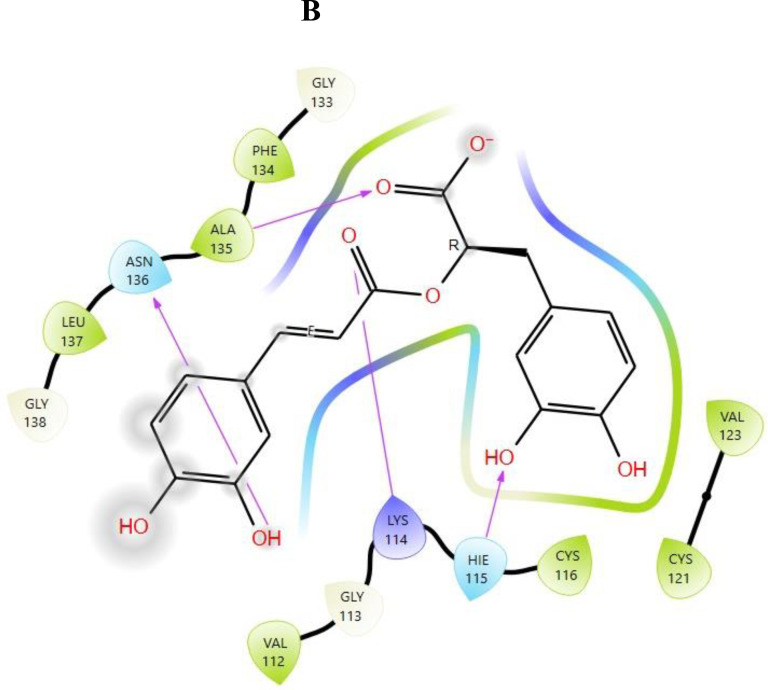	-1.359	HIE 917
5XGJ- IMPHY004597	B 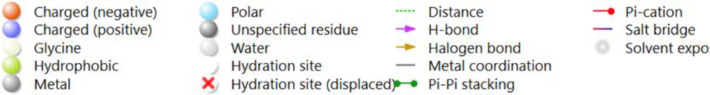	-6.812	ASP 810, TYR 836, VAL 851,

#### 2.8.3 Protein preparation and binding site analysis

The X-ray crystal structures of the proteins (PDB IDs: 1NFK, 5XGJ) were purchased from the Protein Data Bank and processed through the Protein Preparation Wizard in Maestro, which encompasses rigorous refinement steps to resolve atomic clashes, remove water molecules and other stray atoms, and add missing atoms and hydrogens. The binding site of the proteins was identified using the Sitemap tool in Maestro, which allowed for an in-depth exploration of potential therapeutic targets in the PI3K/AKT pathway. These computational approaches provide a solid paradigm to investigate protein interactions and formulate therapeutic approaches in the PI3K/AKT pathway (28).

#### 2.8.4 Molecular docking

The molecular docking was performed using the Glide (Grid-based Ligand Docking with Energetics) protocol as described by Richard A. Friesner et al. (2004). The docking procedure was carried out using a two-step protocol. Initially, ligands were docked using the Standard Precision (SP) mode to screen potential binding conformations. Subsequently, the top-scoring ligand poses were re-docked using the Extra Precision (XP) mode for more rigorous evaluation. The qualified ligand conformations were finally analyzed based on their Glide scores and binding interactions (29).

#### 2.8.5 Molecular dynamic simulation

The compounds were filtered based on several different criteria, including the Glide score (Kcal/mol), protein-ligand non-bonded interactions, ligand complementarity with the active site and relevant literature. The best-ranking complex from the above filtering was closely examined with molecular dynamics (MD) simulations using the Desmond module within the Schrödinger Suite 2022. The primary objective of the MD was to examine intermolecular interactions and the stability of the complexes over time. For system preparation TIP3P solvent model was used, and an orthorhombic water box was chosen with counter ions for neutralization of the system. The prepared system was loaded into the molecular dynamics space for a 200 ns MD simulation. At the end of the MD simulation, the complexes were analyzed using the simulation interaction diagram panel in the Desmond module (30).

#### 2.8.6 Density functional theory calculation

Density Functional Theory (DFT) calculations performed at the B3LYP/6-31G* level revealed that rosmarinic acid has a HOMO energy of −5.546 eV and a LUMO energy of −1.650 eV, with a calculated HOMO–LUMO energy gap of 3.895 eV. The moderate energy gap indicates favorable electronic stability while maintaining sufficient chemical reactivity. The HOMO was predominantly localized over the aromatic and hydroxyl-containing regions, whereas the LUMO was distributed across the conjugated framework, suggesting potential electron donor and acceptor sites that may facilitate intermolecular interactions and contribute to the biological activity of the molecule (31).

## 3 Results

### 3.1 Antioxidant assays

#### 3.1.1 DPPH radical scavenging assay

**Figure 2:**
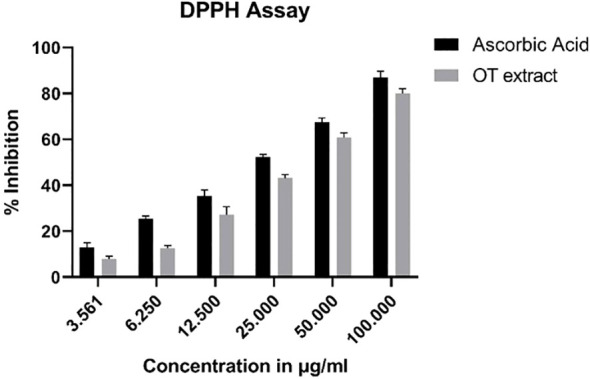
The graph showed that the Ocimum tenuiflorum (OT) extract exhibited DPPH radical scavenging activity, comparable to ascorbic acid, across concentrations ranging from 3.561 to 100 µg/mL.

The DPPH radical scavenging assay demonstrated a concentration-dependent increase in antioxidant activity for both *Ocimum tenuiflorum* (OT) extract and ascorbic acid (3.561–100 µg/mL), with maximum inhibition of 87.8% for ascorbic acid and 82.5% for OT extract, and calculated IC_50_ values of 23.3 µg/mL and 34.7 µg/mL, respectively, indicating stronger antioxidant activity of the standard compared to the extract. (**Figure 2**).

#### 3.1.2 ABTS radical cation decolorization assay

**Figure 3:**
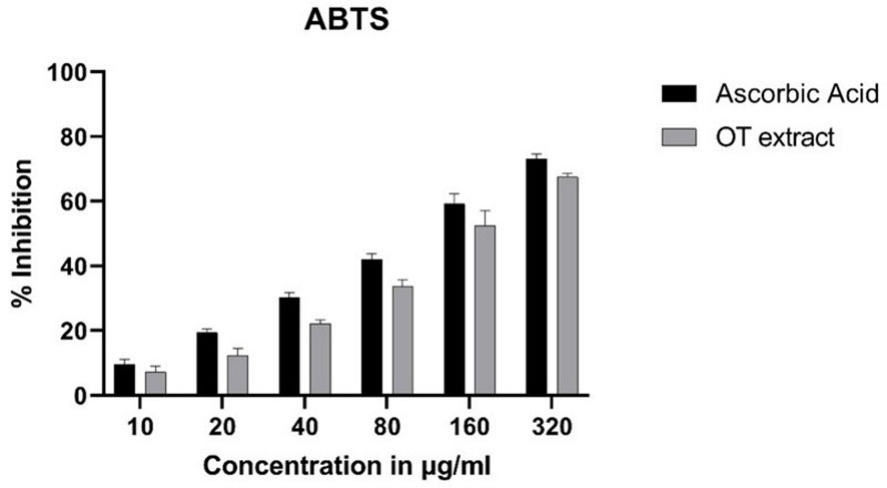
The graph showed that the *Ocimum tenuiflorum* (OT) extract exhibited ABTS radical scavenging activity, comparable to ascorbic acid, across concentrations ranging from 10 to 320 µg/mL, with antioxidant activity expressed as percentage inhibition.

The ABTS radical cation decolorization assay showed a concentration-dependent increase in antioxidant activity for both *Ocimum tenuiflorum* (OT) extract and ascorbic acid (10–320 µg/mL), with maximum inhibition of 73.1% for ascorbic acid and 68.73% for OT extract at 320 µg/mL, indicating that although ascorbic acid exhibited slightly higher scavenging activity, the OT extract also demonstrated substantial antioxidant potential. (**Figure 3**).

#### 3.1.3 Ferric reducing antioxidant power assay

**Figure 4:**
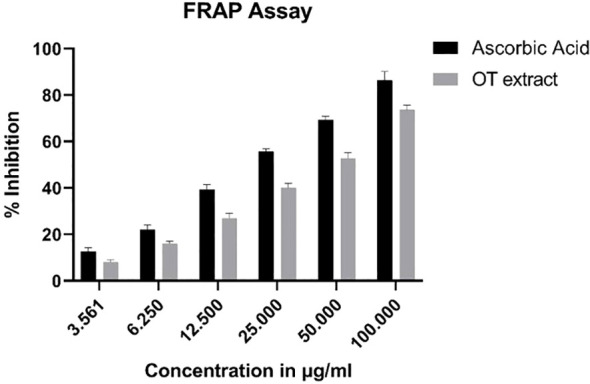
The graph showed that the *Ocimum tenuiflorum* (OT) extract exhibited ferric reducing antioxidant power (FRAP) comparable to ascorbic acid across concentrations ranging from 3.561 to 100 µg/mL.

The Ferric Reducing Antioxidant Power (FRAP) assay demonstrated a concentration-dependent increase in reducing capacity for both *Ocimum tenuiflorum* (OT) extract and ascorbic acid (3.561–100 µg/mL), with maximum activity observed at 100 µg/mL showing 89% inhibition for ascorbic acid and 76% for OT extract, and calculated IC_50_ values of 116.8 µg/mL and 149.2 µg/mL, respectively, indicating a stronger ferric reducing ability of the standard compared with the plant extract. (**Figure 4**).

### 3.2 Anti-inflammatory assays

#### 3.2.1 Nitric oxide assay

**Figure 5:**
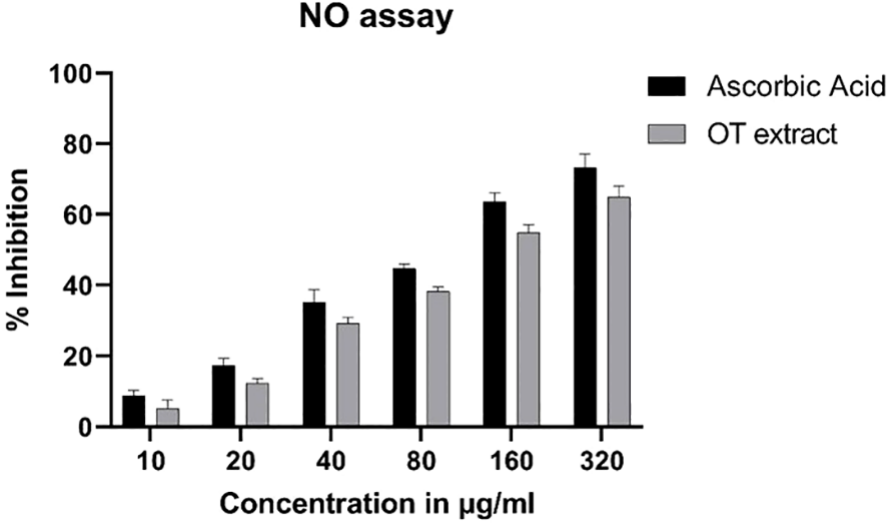
The graph showed that the *Ocimum tenuiflorum* (OT) extract exhibited dose-dependent inhibition of nitric oxide (NO) production in LPS-stimulated RAW 264.7 macrophages, comparable to ascorbic acid, across concentrations ranging from 10 to 320 µg/mL.

The Nitric Oxide (NO) scavenging assay demonstrated a concentration-dependent increase in anti-inflammatory activity for both *Ocimum tenuiflorum* (OT) extract and ascorbic acid (10–320 µg/mL), with maximum inhibition of 73.28% for ascorbic acid and 65% for OT extract at 320 µg/mL, and calculated IC_50_ values of 102.1 µg/mL and 136.4 µg/mL, respectively, indicating stronger nitric oxide scavenging activity of the standard compared with the plant extract. (**Figure 5**).

#### 3.2.2 Cyclooxygenase inhibition assay

**Figure 6:**
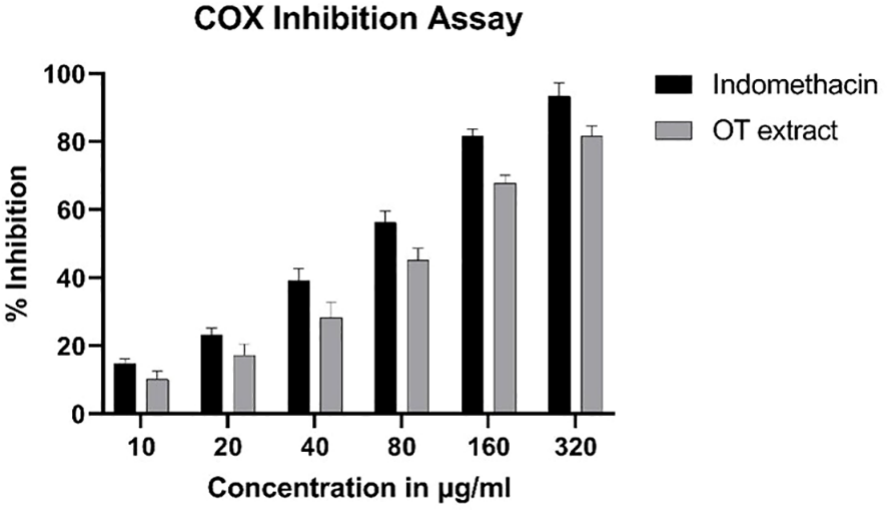
The graph showed that the *Ocimum tenuiflorum* (OT) extract exhibited dose-dependent cyclooxygenase (COX) inhibitory activity, comparable to indomethacin, across concentrations ranging from 10 to 320 µg/mL.

The Cyclooxygenase (COX) inhibition assay demonstrated a concentration-dependent increase in anti-inflammatory activity for both *Ocimum tenuiflorum* (OT) extract and the standard indomethacin (10–320 µg/mL), with maximum inhibition of 93.48% for indomethacin and 81.6% for OT extract at 320 µg/mL, and calculated IC_50_ values of 65.2 µg/mL and 96.6 µg/mL, respectively, indicating stronger COX inhibitory activity of the standard compared with the plant extract. (**Figure 6**).

### 3.3 Cytotoxicity assays

#### 3.3.1 MTT assay

**Figure 7:**
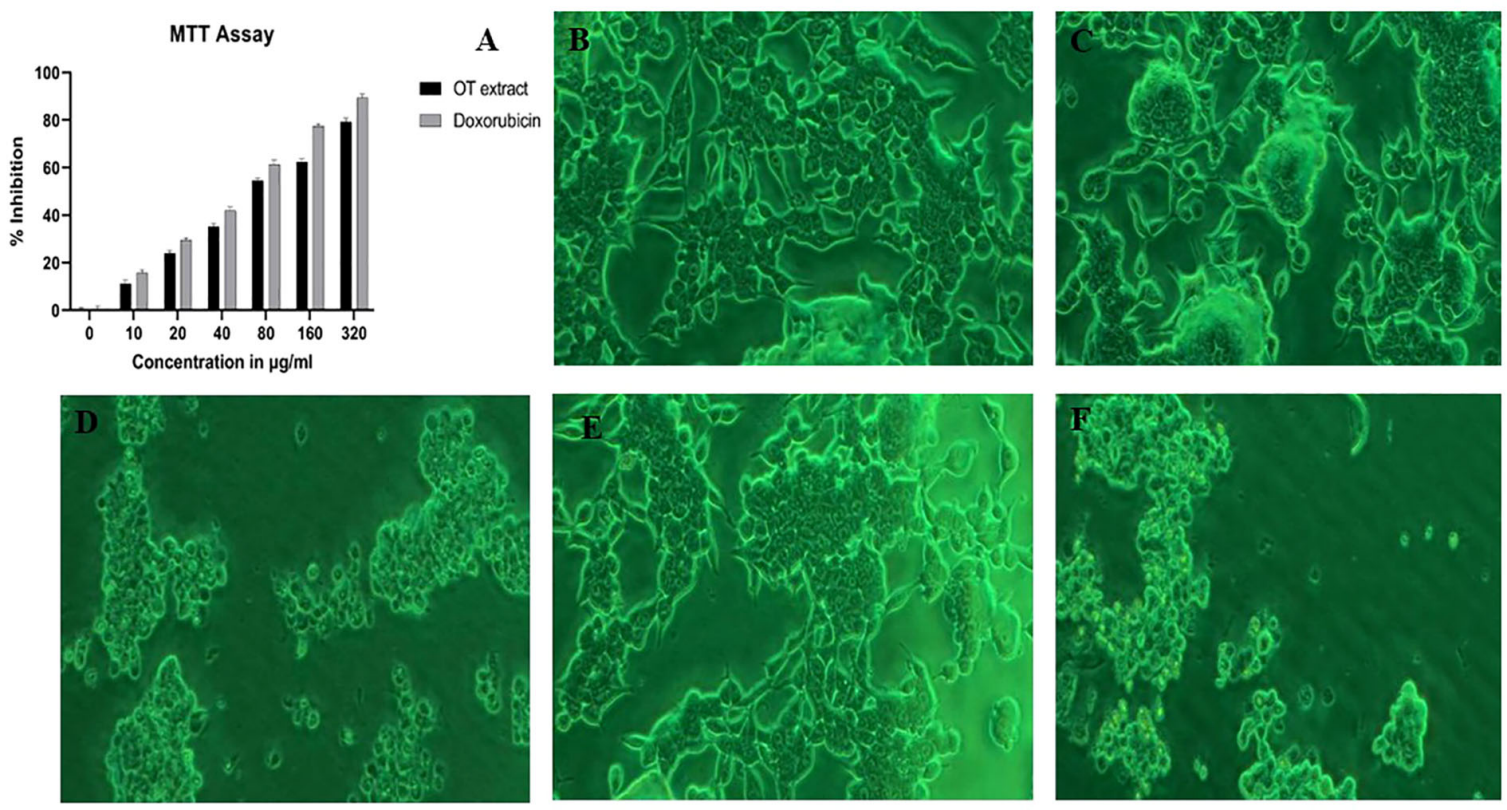
MTT assay demonstrating the cytotoxic effect of *Ocimum tenuiflorum* (OT) extract on HCT116 colorectal cancer cells. **(A)** Bar graph showing the percentage inhibition of HCT116 cell viability following treatment with increasing concentrations of *Ocimum tenuiflorum* (OT) extract and doxorubicin. **(B)** Control, **(C)** Doxorubicin-treated cells (low concentration), **(D)** Doxorubicin-treated cells (high concentration), **(E)** OT extract-treated cells (low concentration), and **(F)** OT extract-treated cells (high concentration).

The MTT assay demonstrated a significant, concentration-dependent increase in cytotoxicity for both *Ocimum tenuiflorum* (OT) extract and doxorubicin within the tested range of 10–320 µg/mL. OT extract produced percentage inhibition values ranging from 11.26 ± 1.45% to 79.25 ± 1.43%, whereas doxorubicin showed comparatively higher inhibition ranging from 15.78 ± 1.23% to 89.36 ± 1.56% (n = 3). At all corresponding concentrations, doxorubicin exhibited greater cytotoxic activity than OT extract. The IC_50_ values were estimated to be approximately 70.4 µg/mL for OT extract and 56.5 µg/mL for doxorubicin. Collectively, these results indicate that although OT extract demonstrates substantial cytotoxic potential, doxorubicin displays relatively higher potency under the experimental conditions. (**Figure 7**).

#### 3.3.2 XTT assay

**Figure 8:**
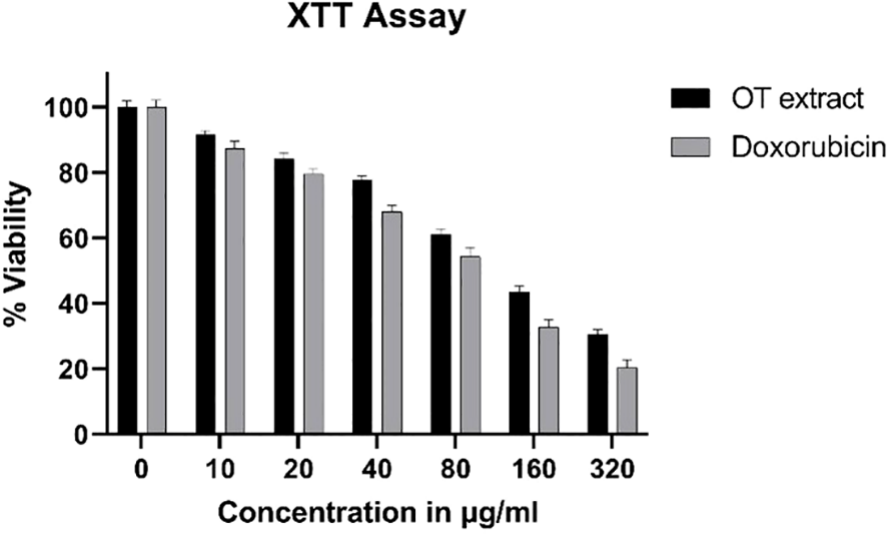
The graph shows a dose-dependent decrease in cell viability following treatment with *Ocimum tenuiflorum* (OT) extract, indicating increasing cytotoxicity with rising concentrations (10–320 µg/mL).

The XTT assay demonstrated a concentration-dependent decrease in HCT116 cell viability following treatment with *Ocimum tenuiflorum* extract and doxorubicin (0–320 µg/mL), with viability declining from 100% to 30.61 ± 1.47% and 20.3 ± 2.5%, respectively (n = 3), and corresponding IC_50_ values of approximately 130.6 µg/mL for OT extract and 96.0 µg/mL for doxorubicin, indicating greater cytotoxic potency of doxorubicin. (**Figure 8**).

#### 3.3.3 LDH release assay

**Figure 9:**
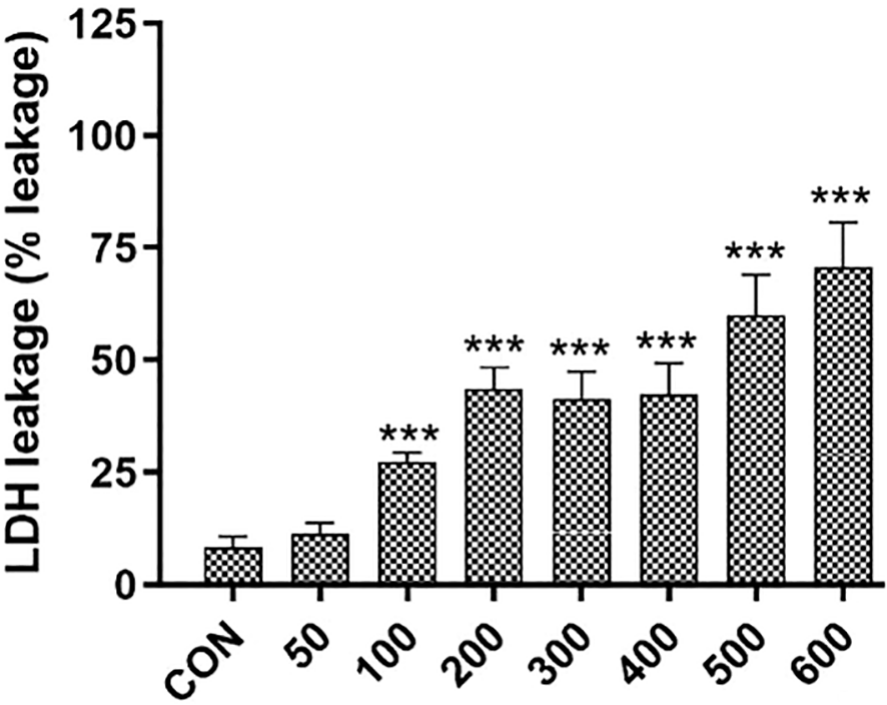
The bar graph shows the percentage of LDH leakage following treatment with increasing concentrations (50–600 µg/mL) of OT extract. LDH leakage increased in a concentration-dependent manner, indicating enhanced membrane damage and cytotoxicity in HCT116 cells. Data are presented as mean ± SD from three independent experiments. ***P < 0.001 compared with the control group.

The Lactate Dehydrogenase Release Assay demonstrated that treatment with *Ocimum tenuiflorum* (OT) extract induced significant membrane damage in HCT116 cells. As shown in **Figure 9**, LDH leakage increased with increasing concentrations of OT extract. The control cells exhibited minimal LDH release (~8–10%), whereas treatment with 50 µg/mL resulted in a slight increase (~10–12%). A significant elevation in LDH leakage was observed from 100 µg/mL onward (p < 0.001), reaching approximately 27% at 100 µg/mL, ~40–45% between 200–400 µg/mL, ~60% at 500 µg/mL, and nearly 70–75% at 600 µg/mL. These findings indicate that OT extract progressively compromises cellular membrane integrity, leading to increased LDH release and suggesting strong cytotoxic activity against HCT116 cells. (**Figure 9**).

### 3.4 Cell viability assays

#### 3.4.1 Trypan blue exclusion assay

**Figure 10:**
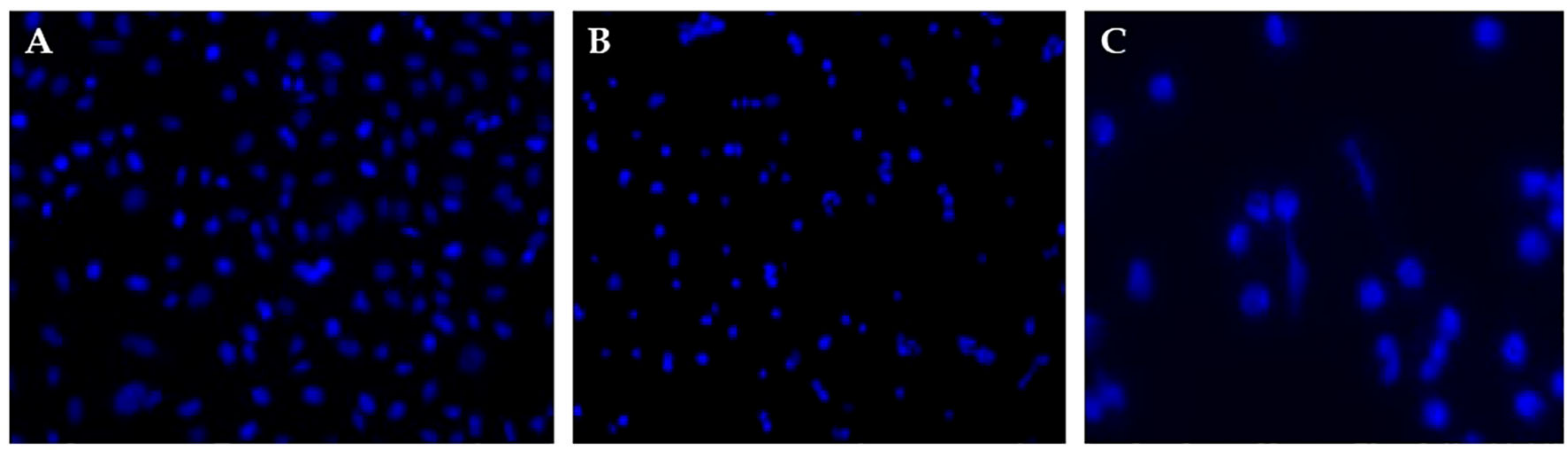
The images indicate that treatment with *Ocimum tenuiflorum* (OT) extract significantly reduced the viability of HCT116 cells. **(A)** Control, **(B)** OT extract [Low concentration], **(C)** OT extract [High concentration].

The results demonstrated a significant reduction in cell viability in HCT116 cells treated with *Ocimum tenuiflorum* (OT) extract. The images (**Figure 10**) reveal an increase in the number of blue-stained (non-viable) cells with higher concentrations of OT extract, indicating enhanced cell death. This visual evidence supports the extract’s potent cytotoxic effect on HCT116 cells, corroborating its potential as an anticancer treatment. (**Figure 10**).

### 3.5 Computational analysis

#### 3.5.1 Protein–protein interaction network analysis of NFKB1

**Figure 11:**
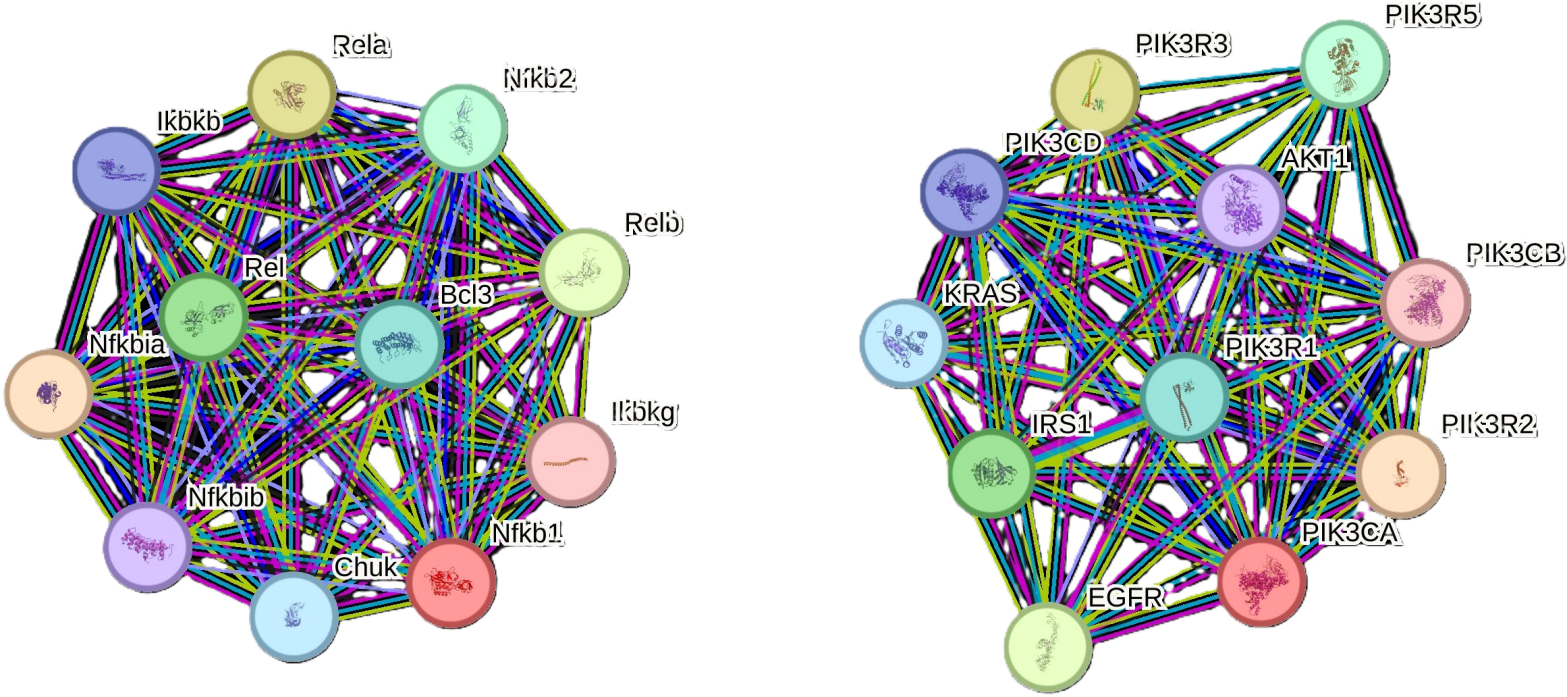
**(A)** Protein–protein interaction (PPI) network of the 1NFK protein generated using the STRING database, **(B)** Protein–protein interaction (PPI) network of the 5XGJ protein generated using the STRING database.

An examination of the STRING’s PPI network showed 11 nodes and 55 edges, with an average node degree of 10. In this highly interconnected network, each protein interacts with an average of ten other proteins, demonstrating the critical role of NFKB1 in this signaling network. Important interaction partners include key players in the NF-κB signaling cascade, including RELA, RELB, REL, NFKB2, NFKBIA, NFKBIB, BCL3, CHUK, IKBKB, and IKBKG. The tight interconnection suggests a robust signaling route that is critical for immune response, inflammation, and cell survival. (**Figure 11A**).

#### 3.5.2 Protein–protein interaction network analysis of PIK3CA

An examination of the STRING’s PPI network showed 11 nodes and 54 edges, with an average node degree of 10. In this highly interconnected network, each protein interacts with an average of ten other proteins, demonstrating the critical role of PIK3CA in this signaling network. Important interaction partners include key players in the PI3K/AKT signaling cascade, including AKT1, PIK3R1, PIK3R2, PIK3CB, PIK3CD, PIK3R3, PIK3R5, IRS1, KRAS, and EGFR. The tight interconnection suggests the progression of cancer and controls cell proliferation and survival. interconnectedness of the PPI network illustrates the central role of PIK3CA in oncogenic signaling, and underscores the potential role of PIK3CA as a therapeutic target. (**Figure 11B**).

#### 3.5.3 Molecular docking

Molecular docking was performed to comparatively evaluate the binding affinities and interaction profiles of all major bioactive constituents of *Ocimum tenuiflorum*. Against two CRC-relevant target proteins. Tumor Necrosis Factor-alpha (PDB ID: 1NFK) and Janus Kinase 1 (PDB ID: 5XGJ). This comparative screening approach was employed to objectively identify the lead bioactive compound based on computational evidence. Among all compounds screened, rosmarinic acid (IMPHY004597) consistently exhibited the highest binding affinity against both target proteins, with docking scores of −5.898 kcal/mol against 1NFK and −6.812 kcal/mol against 5XGJ substantially outperforming other bioactive constituents at both targets. This computational superiority, combined with rosmarinic acid’s established capacity to simultaneously suppress NF-κB and JAK-STAT signaling pathways critically dysregulated in CRC provides a robust, evidence-based validation for its identification as the principal bioactive contributor in *O. tenuiflorum* and its subsequent prioritization for molecular dynamics simulation and DFT analysis.

The significant interactions were with ASN136, ALA135, LYS114 and HIE115 in the 1NFK-Rosmarinic acid complex and ASP810, TYR836 and VAL851 in the 5XGJ-Rosmarinic acid complex. These residues are important for ligand binding and likelihood of structural stability of the overall protein-ligand complex. Based on the docking scores and interactions of Rosmarinic acid compared to all other compounds from *Ocimum tenuiflorum*, it could serve as potential lead compound for further development. (**Table 1**).

#### 3.5.4 Molecular dynamic simulation

**Figure 12:**
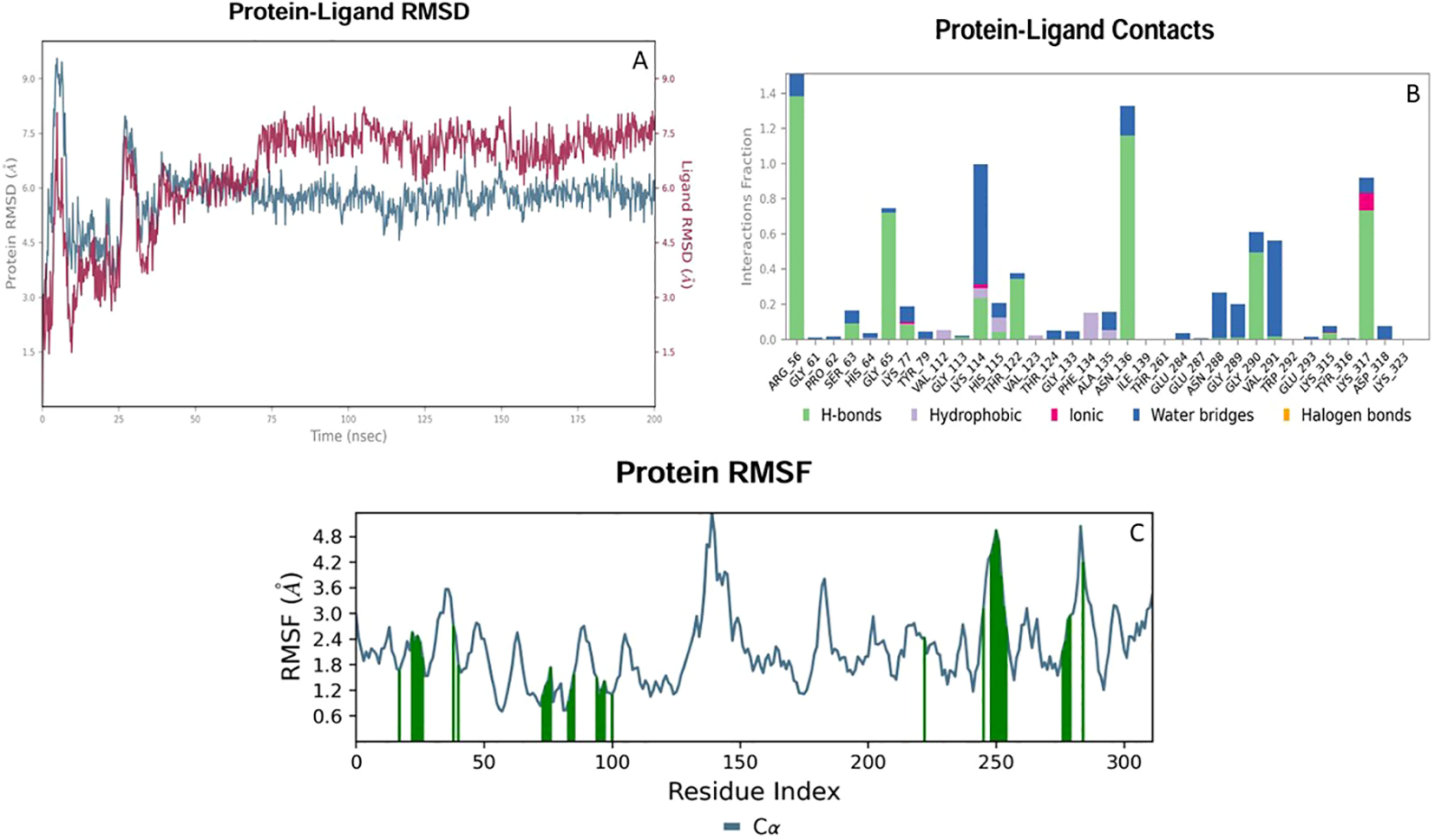
**(A)** RMSD graphs of Receptor-ligand complexes in MD simulations, **(B)** Protein-ligand contacts, **(C)** Root mean square fluctuation (RMSF) profile of the protein–rosmarinic acid complex.

A simulation study involved molecular dynamics (MD) to determine the stability of the receptor-ligand complex, validate the computed ligand binding mode, and inspect for any possible interactions, which was confirmed through Glide XP docking. In this study, we performed a 200 ns MD simulation of the 1NFK - IMPHY004597 complex. The benefit of performing a 200 ns MD simulation, is that it was long enough to observe the molecular and atomic level conformational changes that are required to observe the stability of the proteins and the dynamic behavior of the ligand. We conducted the 200 ns MD simulation in Desmond, which is part of the Schrödinger suite. In order to view the trajectories to understand deviation fluctuation and intermolecular interactions we used the simulation interaction diagram panel. We obtained the root mean square deviation (RMSD) to view the deviation of the protein backbone throughout the 200 ns MD simulation time. The RMSD plot (**Figure 12A**) displays the time evolution of the backbone atoms of the 1NFK protein and the heavy atoms of the rosmarinic acid ligand throughout the 200 ns simulation time. After an initial equilibration time (~10 ns), the protein backbone RMSD stabilized and fluctuated within ± 2.7 Å, clearly indicating a structural stable complex for throughout the entire simulation. The ligand-RMSD exhibited moderate fluctuations between ~1.2 Å to ~2.2 Å, well within the limits of acceptable binding, and with no indication of displacement and consistent binding in the binding pocket. The RMSD data illustrate that the 1NFK– rosmarinic acid complex preserved good structural stability over the duration of the simulation. Protein ligand contacts (**Figure 12B**) are shown as the time-resolved protein–ligand interaction profile for the MD simulation. Strong and persistent interactions between the ligand and important residues within the binding pocket were observed. These interactions involved hydrogen bonds, hydrophobic contacts, water bridges, and π–π stacking interactions. Our RMSF plot demonstrated that both terminal regions of the protein exhibited greater fluctuations compared to the intermediate central region of the protein, which exhibited stable and consistent structure residue-by-residue. This is an expected finding because both terminal regions are expected to be relatively flexible and exposed to solvent. The secondary structural constituents (alpha-helices and beta-strands) exhibited markedly lower RMSF, indicating that they were conformationally stable throughout the simulation. The structured regions are shown from the simulations in the diagrams as red (helices) or blue (beta-strands) and they persisted for over 70% of the simulation indicating that they were relatively rigid and maintained the integrity of the structure. The regions involved in binding, and shown in green in the figure also exhibited only slightly greater fluctuation which supports that the ligand-hosted complex was structurally sturdy. The residues are considerable rigid while bound to rosmarinic acid and contributed to localization steadfastness of the protein structure. The overall RMSF data suggests that all three scenarios maintained stable conformation with recognizable flexible terminus, the most crucial structural and functional components of the protein were maintained. There were no significant structural or functional transitions in the protein throughout the simulation results in stable binding interaction was reliable and robust throughout the simulation (**Figures 12A–C**).

#### 3.5.5 Density functional theory calculation

**Figure 13:**
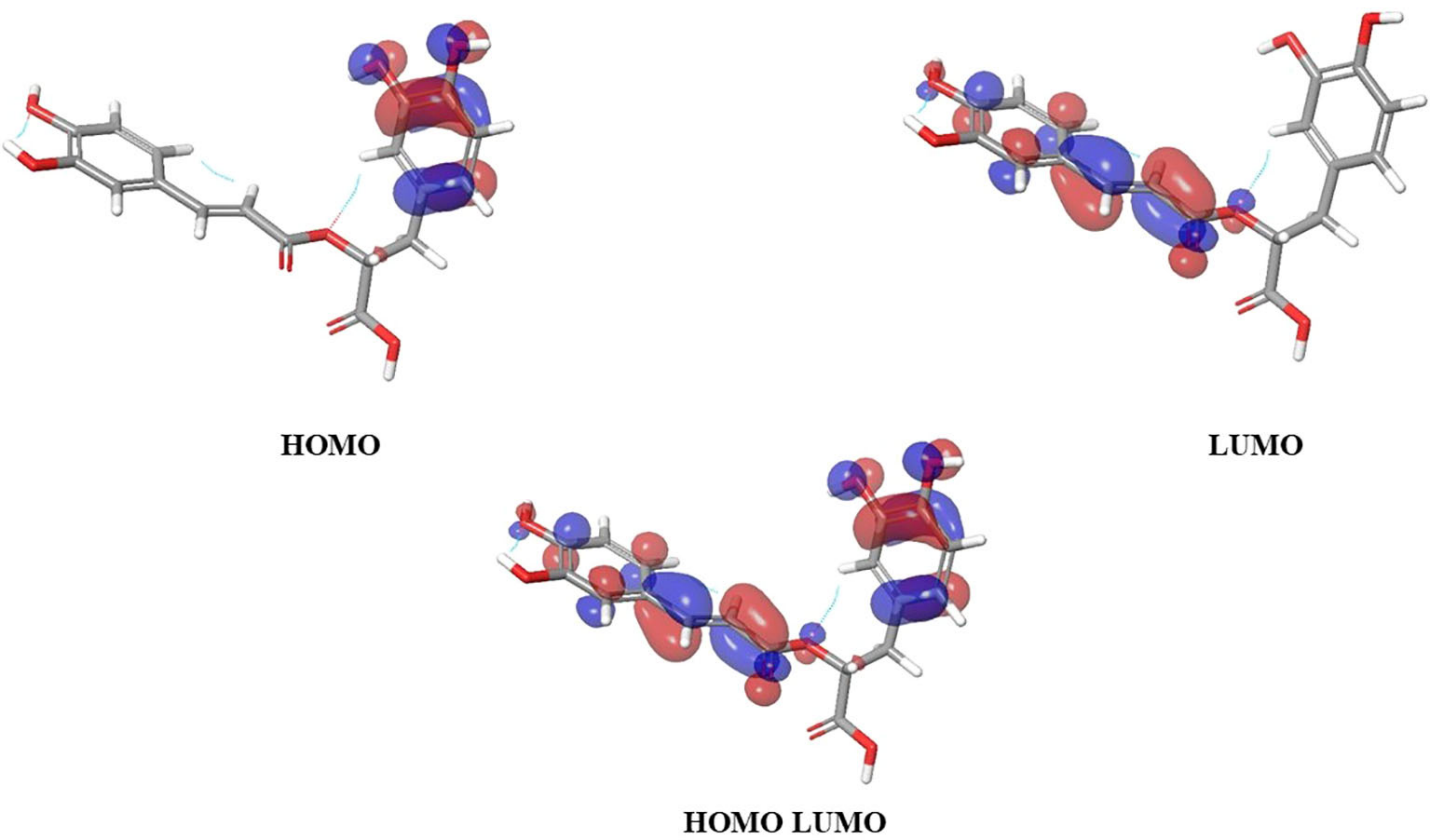
Representation of HOMO, LUMO, and HOMO LUMO in compound (IMPHY004597).

Density Functional Theory (DFT) calculations performed at the B3LYP/6-31G level revealed that rosmarinic acid has a HOMO energy of −5.546 eV and a LUMO energy of −1.650 eV, with a calculated HOMO–LUMO energy gap of 3.895 eV. The moderate energy gap indicates favorable electronic stability while maintaining sufficient chemical reactivity. The HOMO was predominantly localized over the aromatic and hydroxyl-containing regions, whereas the LUMO was distributed across the conjugated framework, suggesting potential electron donor and acceptor sites that may facilitate intermolecular interactions and contribute to the biological activity of the molecule. (**Figure 13**).

## 4 Discussion

The *Ocimum tenuiflorum* ethanolic extract demonstrated significant, concentration-dependent antioxidant activity across three mechanistically distinct assay systems hydrogen atom transfer (DPPH; IC_50_: 34.7 µg/mL), electron transfer (ABTS; maximum inhibition: 68.73% at 320 µg/mL), and metal reduction (FRAP; IC_50_: 149.2 µg/mL), with inter-assay concordance confirming broad-spectrum radical scavenging capacity rather than assay-specific artefact (10, 22). These findings are consistent with the established polyphenolic composition of *O. tenuiflorum* and are biologically relevant to CRC, wherein ROS-driven NF-κB and STAT3 activation, iNOS/COX-2 upregulation, and oxidative DNA damage collectively sustain the adenoma-carcinoma transition (4, 5). The observed antioxidant activity thus implicates a plausible chemopreventive mechanism operating upstream of the inflammatory and proliferative pathways central to CRC pathogenesis, consistent with evidence for curcumin, resveratrol, and epigallocatechin gallate in preclinical CRC models (15). Cytotoxic activity against HCT116 cells was confirmed across four complementary assays (MTT, XTT, LDH release, Trypan Blue exclusion), each interrogating a distinct parameter of cell death, with cross-assay concordance substantiating the robustness of these findings. PPI network analysis of NFKB1 (11 nodes, 55 edges) and PIK3CA (11 nodes, 54 edges) revealed high-connectivity signaling hubs integrating inflammatory, survival, and proliferative inputs including RELA, IKBKB, AKT1, KRAS, and EGFR reinforcing the therapeutic rationale for dual NF-κB and JAK-STAT targeting in CRC (4, 5).

Molecular docking identified rosmarinic acid as the lead bioactive constituent, achieving superior binding affinities at both TNF-α/1NFK (−5.898 kcal/mol) and JAK1/5XGJ (−6.812 kcal/mol) relative to all other screened phytochemicals and the reference standard doxorubicin (−3.286 and −1.359 kcal/mol, respectively) (15). The stability of the rosmarinic acid–1NFK complex was confirmed over 200 ns MD simulation, with backbone RMSD stabilizing within ±2.7 Å and ligand RMSD fluctuating between 1.2–2.2 Å, well within the ≤3.0 Å threshold for productive complex stability (15). DFT analysis further validated drug-likeness, with a HOMO–LUMO gap of 0.14314 a.u. (3.895 eV) indicating enhanced electronic polarizability and reactivity, and a predominantly electron-rich MEP surface (mean ESP: −0.76 kcal/mol) consistent with favorable electrostatic complementarity at the LYS114 and HIE115 residues of 1NFK (30).

The integrated findings support a mechanistic rationale for the adjunctive use of O. tenuiflorum extract alongside FOLFOX and FOLFIRI chemotherapy regimens in advanced CRC. A critical mechanism of 5-FU resistance involves chemotherapy-induced NF-κB activation, which upregulates anti-apoptotic effectors (Bcl-2, Bcl-xL, survivin) and promotes epithelial-to-mesenchymal transition (32). Rosmarinic acid’s demonstrated capacity for NF-κB suppression corroborated by docking, MD simulation, and the independent experimental findings of Liu et al. (2025) provides a compelling basis for its potential role as a chemosensitizer capable of lowering effective therapeutic doses and attenuating treatment-related toxicity (15, 32). This is further supported by Ballesteros-Tapias et al. (2024), who demonstrated rosmarinic acid’s capacity to reverse chemoresistance and confer chemoprotection in experimental cancer models (16). Translational application of these findings will nevertheless require resolution of pharmacokinetic challenges associated with rosmarinic acid, including rapid gastrointestinal absorption, extensive hepatic first-pass metabolism, gut microbiota-mediated biotransformation, and a short effective plasma half-life that may preclude sustained NF-κB-inhibitory concentrations at conventional oral doses (16). Nanoformulation strategies including polymeric nanoparticles, solid lipid nanoparticles, and cyclodextrin inclusion complexes represent established approaches for enhancing bioavailability, extending plasma residence time, and achieving targeted colonic delivery, and should be systematically evaluated in future preclinical studies (14, 16). Definitive dosage recommendations and pharmacodynamic characterization in relevant in vivo models constitute essential next steps in the translational development of *O. tenuiflorum* as a phototherapeutic candidate for colorectal cancer.

The present study has several limitations. Phytochemical characterization relied on IMPPAT database structures rather than direct HPLC or LC-MS profiling of the crude extract, precluding confirmation of individual constituent concentrations in the test preparation. Second, *in vitro* experiments were restricted to the MSI-H HCT116 cell line, limiting generalizability to the MSS-predominant CRC population, future validation in HT29 and SW480 cell lines is warranted. The whilst suppression of NO production and COX activity in RAW 264.7 macrophages suggest potential modulation of TME-associated inflammatory signaling, confirmation in CRC-relevant co-culture or TAM polarization models is required. Molecular docking and dynamics findings represent computationally hypothesized interactions pending experimental validation through target-specific functional assays. These limitations define a clear framework for future preclinical investigation of *O. tenuiflorum* and rosmarinic acid in colorectal cancer.

## 5 Conclusion

This study thoroughly assessed the anticancer capability of *Ocimum tenuiflorum* (Holy Basil) against colorectal cancer (CRC) *in vitro* and *in silico*. The ethanolic extract of *O. tenuiflorum* displayed strong cytotoxic, antioxidant, and anti-inflammatory effects in HCT116 colorectal cancer cells. The cytotoxicity of the extract was shown to be effective in cytotoxicity assays (MTT, LDH, and XTT assays) as determined by the spectacular inhibition of cancer cell viability (trypan blue exclusion assay) and the ability of the extract to induce membrane damage in the CRC cell line. Antioxidant assays (DPPH, ABTS, FRAP) demonstrated strong radical scavenging abilities, while anti-inflammatory assays demonstrated a significant inhibition of nitric oxide and cyclooxygenase, suggesting *O. tenuiflorum* extracts potentially modulate inflammation and oxidative stress which are two major contributors to the progression of CRC. Molecular docking studies against the proteins involved in the PI3K/Akt and MAPK pathways (PDB ID: 1NFK and PDB ID: 5XGJ) reveal that rosmarinic acid was the only compound with the highest binding affinity with the targeted protein with the docking scores of -5.898 and -6.812 kcal/mol, respectively. Analysis of the interactions was even more promising in that the binding mode exhibited strong hydrogen bonds and some hydrophobic interactions with key active site residues and the standard reference compound had no hydrogen bonds and weak hydrophobic interactions. In this study the rosmarinic acid-protein complexes structure took place within acceptable molecular dynamics (MD) congruity parameters over the entire 200 ns MD simulation as the RMSD and RMSF plots of the rosmarinic acid-protein complex had remained tight and showed that they were stable. Density functional theory (DFT) analysis revealed that rosmarinic acid exhibits high electronic reactivity, which supports its drug-likeness and confirms its molecular stability. This study concludes that *Ocimum tenuiflorum* and specifically rosmarinic acid, is the natural candidate for colorectal cancer treatment. The significant activity on computational and experimental platforms is outstanding endorsement for further preclinical and clinical studies to determine safety and efficacy for *Ocimum tenuiflorum* and rosmarinic acid, possibly as an adjunctive cancer treatment.

## Data Availability

The raw data supporting the conclusions of this article will be made available by the authors, without undue reservation.
